# Anti-apolipoprotein A-1 IgG, incident cardiovascular events, and lipid paradox in rheumatoid arthritis

**DOI:** 10.3389/fcvm.2024.1386192

**Published:** 2024-05-20

**Authors:** Denis Mongin, Sabrina Pagano, Celine Lamacchia, Catherine Juillard, Paola Antinori-Malaspina, Diana Dan, Adrian Ciurea, Burkhard Möller, Cem Gabay, Axel Finckh, Nicolas Vuilleumier

**Affiliations:** ^1^ Division of Rheumatology, Geneva University Hospital and Faculty of Medicine, University of Geneva, Geneva, Switzerland; ^2^ Division of Laboratory Medicine, Department of Diagnostics and of Medical Specialties, Geneva University Hospitals and Geneva University, Geneva, Switzerland; ^3^ Division of Rheumatology, Lausanne University Hospital and Faculty of Medicine, University of Lausanne, Lausanne, Switzerland; ^4^ Division of Rheumatology, Zurich University Hospital and Faculty of Medicine, University of Zurich, Zurich, Switzerland; ^5^ Division of Rheumatology and Immunology, Bern University Hospital and Faculty of Medicine, University of Bern, Bern, Switzerland

**Keywords:** anti-apolipoprotein A-1 IgG, autoantibodies, cardiovascular disease, major adverse cardiovascular events, rheumatoid arthritis

## Abstract

**Objective:**

To validate the prognostic accuracy of anti-apolipoprotein A-1 (AAA1) IgG for incident major adverse cardiovascular (CV) events (MACE) in rheumatoid arthritis (RA) and study their associations with the lipid paradox at a multicentric scale.

**Method:**

Baseline AAA1 IgG, lipid profile, atherogenic indexes, and cardiac biomarkers were measured on the serum of 1,472 patients with RA included in the prospective Swiss Clinical Quality Management registry with a median follow-up duration of 4.4 years. MACE was the primary endpoint defined as CV death, incident fatal or non-fatal stroke, or myocardial infarction (MI), while elective coronary revascularization (ECR) was the secondary endpoint. Discriminant accuracy and incidence rate ratios (IRR) were respectively assessed using C-statistics and Poisson regression models.

**Results:**

During follow-up, 2.4% (35/1,472) of patients had a MACE, consisting of 6 CV deaths, 11 MIs, and 18 strokes; ECR occurred in 2.1% (31/1,472) of patients. C-statistics indicated that AAA1 had a significant discriminant accuracy for incident MACE [C-statistics: 0.60, 95% confidence interval (95% CI): 0.57–0.98, *p* = 0.03], mostly driven by CV deaths (C-statistics: 0.77; 95% CI: 0.57–0.98, *p* = 0.01). IRR indicated that each unit of AAA1 IgG increase was associated with a fivefold incident CV death rate, independent of models’ adjustments. At the predefined and validated cut-off, AAA1 displayed negative predictive values above 97% for MACE. AAA1 inversely correlated with total and HDL cholesterol.

**Conclusions:**

AAA1 independently predicts CV deaths, and marginally MACE in RA. Further investigations are requested to ascertain whether AAA1 could enhance CV risk stratification by identifying patients with RA at low CV risk.

## Introduction

Patients with autoimmune diseases (AID), including rheumatoid arthritis (RA), exhibit a 1.4- to 3.6-fold increased risk of cardiovascular (CV) diseases, independent of traditional CV risk factors (1, 2). Accelerated inflammation-driven atherosclerosis in RA is considered to result from the cumulative effects of traditional, non-traditional CV risk factors, and genetic predispositions (3). As a result, usual CV risk stratification tools (Framingham Risk Score, modified SCORE, Pooled Cohort Risk Equation) used for screening purposes in the general population have repeatedly been shown to substantially underestimate CV risk in RA (4), even after the application of the 1.5 multiplication correction factor proposed by the European League Against Rheumatism (EULAR) (5). Such sub-optimal performance is believed to partially result from the combination of the lipid paradox in RA consisting of lower total high- and low-density lipoprotein (HDL and LDL) cholesterol levels and a high pro-atherogenic indexes (total cholesterol to HDL ratio and triglyceride to HDL ratio) due to systemic inflammation, together with other RA-specific factors that are not adequately factored in the current CV risk stratification models in RA (6).

Because the prediction of major adverse CV events (MACE) is an unmet clinical need in RA management, numerous initiatives have been undertaken to optimize CV risk stratification in RA using various models combining clinical features, radiological, and/or biomarkers. Unfortunately, proposed models of cardiovascular risk predictions have only resulted in moderated discriminative capacity, with areas under the curve (AUC) in the range of 0.70–0.80, which is why further improvements are deemed necessary (7–17).

In RA, several autoantibodies easily measurable in the blood have shown some promise as alternative surrogate CV risk markers (7, 14, 15). Among these, the class of autoantibodies against HDL is under active scrutiny. Among the different components of humoral response against HDL particles (16), autoantibodies against anti-apolipoprotein A-1 (AAA1)—the major protein fraction of HDL molecules conferring to the latter most of their atheroprotective functions—have been the most extensively studied and characterized (16). Large multicenter prospective studies have demonstrated in non-AID patients that AAA1 represents an independent CV risk factor (18), predicting incident CV events and death despite being associated with a favorable lipid profile (except decreased HDL levels) (19–22), reminiscent of the lipid paradox in RA. Smaller single-center studies indicated that AAA1 were associated with histological features, such as atherosclerotic plaque vulnerability and burden in humans (23, 24). Experimental data indicate that AAA1 elicit pro-arrhythmogenic and pro-inflammatory responses through specific innate immune receptor complex signaling, generating systemic inflammation, foam cell formation (25–28), atherosclerosis, atherothrombosis, myocardial necrosis, and death in mice (23, 29–31). Three case-control studies showed that anti-HDL antibodies are raised in RA, where they associated with lower HDL levels and antioxidant function, higher systemic inflammatory profile, and increased occurrence of carotid atherosclerotic plaques (32, 33). To date, only one single-center study of limited size in RA suggested that AAA1 could not only independently predict MACE at 9 years, but also improve the 10-year predictive ability of the Framingham Risk Score (14).

Therefore, we performed a phase III biomarker study to (i) challenge the prognostic value of AAA1 for incident MACE on a large multicenter scale and (ii) explore their relationship with the RA lipid paradox, using the Swiss Clinical Quality Management (SCQM) registry enrolling patients from private practices and academic and non-academic centers all over Switzerland.

## Material and methods

### Ethical approval

The study protocol was approved by the local ethics committee of the University Hospital of Geneva (PB_2018-00317), the SCQM Biobank Scientific Advisory Board, and SCQM Foundation board. All participants gave informed consent before enrollment in accordance with the Declaration of Helsinki.

### Patient and public involvement

Neither the patient nor the public was directly involved in this study. All research projects with the SCQM registry are critically reviewed by a panel that includes several patient representatives.

### Study population

We conducted a prospective cohort study, consisting of 1,472 patients with RA included in the SCQM (www.SCQM.ch) registry, for which a serum sample was available in the SCQM biobank. Briefly, the SCQM registry was founded in 1997 and enrolls patients experiencing inflammatory rheumatic diseases (IRD), such as RA, axial ankylosing spondylarthritis, and psoriasis arthritis, originating from private practices (50%), academic centers (20%), and non-academic centers (30%), providing a real-life IRD population sample of Switzerland. Longitudinal data collection, including cardiovascular events and associated risk factors encompassing the period before and after inclusion in the register, is provided by treating rheumatologists through dedicated case report forms (CRF). Assessments are performed at regular intervals, approximately 1–4 times per year (disease activity, anti-rheumatic treatments, side effects, reasons for discontinuation, comorbidities, etc.).

### Samples and biochemical analyses

The samples were processed and stored at −80°C until analysis. AAA1 were measured using an extensively validated in-house Enzyme Linked Immunosorbent Assays (ELISA) protocol (18–24, 28). The conventional AAA1 seropositivity cut-off was prospectively defined and usually set at an optical density measured at 405 nm (OD405) > 0.64 arbitrary unit (AU), corresponding to the 97.5th percentile of AAA1 levels obtained from healthy blood donors (18–24, 28).

Cholesterol HDL, non-HDL, and LDL were measured using standard chemistry assays (Roche 8000/H Cobas) whereas LDL cholesterol values were calculated using the Friedewald formula. The conventional atherogenic indexes consisting of total cholesterol to HDL ratio, and triglycerides to HDL ratio were generated. As the biochemical gold standard for cardiac ventricular strain and of myocardial injury, N-terminal pro-brain natriuretic peptide (NT-proBNP) and high-sensitive cardiac troponin T (hs-cTnT) were respectively measured using electrochemiluminescence methods on routine auto-analyzers (Elecsys™, Roche, Switzerland).

### Definition of study outcomes

Three prespecified outcomes were considered for this study. The primary composite outcome of MACE was defined as non-fatal myocardial infarction (MI), or non-fatal stroke or CV death (including fatal stroke and/or myocardial infarction) (22, 34). The second endpoint was the need for elective coronary revascularization (ECR). Endpoints were either reported by the treating rheumatologist in the SCQM registry, by a study nurse, or by the patient using a specific self-reported questionnaire (35). In case of inconsistent responses between the patient, the treating physician, or the rheumatologist, the study coordinators, blinded to AAA1 results, adjudicated the final diagnosis (36). Only events confirmed by medical records, CRFs, and physicians were taken into account.

The third outcome consisted of pro-atherogenic indices, such as the total cholesterol to HDL ratio and the triglyceride to HDL ratio (6).

### Statistical analyses

Differences in the proportion of categorical variables were tested using the chi-square test with Yates correction when adequate. Differences for continuous variables were tested using the *t*-test, and with the Mann–Whitney *U*-test for the variables with a non-normal distribution. Spearman’s rank test was used for correlation analyses. C-statistics using continuous AAA1 OD values were used to generate the AUC, as well as sensitivity (SE), specificity (SP), and positive and negative predictive values (PPV and NPV, respectively), as well as positive and negative likelihood ratios (LR+ and LR−, respectively). Cumulative incidence graphs were used to visually depict the occurrence of MACE and its components over time, allowing us to estimate and compare the cumulative incidence rates between AAA1 seropositive and seronegative patients. The log-rank test was employed to assess the statistical significance of survival differences observed among these strata. Incidence of MACE, its components, and ECR were calculated as the number of events per 1,000 patient years.

Incidence rate ratios (IRR) between AAA1 seropositive and seronegative patients, or for each increase of 1 AU of AAA1, were estimated using Poisson regression. To account for potential confounding factors, the regressions were also performed on a matched dataset. Patients with positive outcomes were matched 1:3 on a population with negative outcomes using propensity scores for each outcome [MACE for the main outcome, its individual components (MI, non-fatal stroke, or CV death), or ECR]. The propensity score was generated using multivariable logistic regression on the following baseline variables: age; disease duration; sex; smoking; past statin use; past hypertension; baseline corticosteroid and conventional synthetic disease-modifying antirheumatic drug (csDMARD) use; NT-proBNP; hs-cTnT; DAS28; HDL cholesterol; and the atherogenic indexes (total cholesterol to HDL ratio and triglyceride to HDL ratio). Missing covariates were handled with multiple imputation with chained equations, using 50 samples and 10 iterations and all covariates in the model.

For the sensitivity analyses, univariate Cox proportional hazards models were performed on the entire dataset and on the matched dataset.

Results are reported with their corresponding 95% confidence intervals (CI). *p*-values <0.05 were considered significant. All analyses were performed using R software (V.4.0.4) (37).

## Results

### Population description

The patients’ characteristics are presented in Table 1. Among the 1,472 patients considered in this study, the AAA1 seropositivity rate was 16% (236/1,472). As shown in Table 1, AAA1 seropositive patients were more likely to be male and treated with TNFi. AAA1 seropositivity was associated with lower median total, HDL, and non-HDL cholesterol levels, as well as a higher rate of CV death when compared to AAA1 seronegative individuals. No other baseline demographic or biomarker difference was observed between these two groups.

**Table 1:** Sociodemographic factors, number of outcomes, and biochemical measures stratified between ApoA1 positive and negative patients.

Number of patients	Overall	AAA1 seronegative	AAA1 seropositive	*p*	Missing data in %
	1,472	1,236	236		
Sociodemographic					
Age, mean (SD)	58.12 (13.25)	58.12 (13.16)	58.10 (13.73)	0.977	0.0
Male sex, *n* (%)	372 (25.3)	294 (23.8)	78 (33.1)	0.004	0.0
Disease duration, mean (SD)	10.68 (9.97)	10.56 (9.85)	11.33 (10.54)	0.280	0.9
Smoker, *n* (%)	393 (26.7)	334 (27.0)	59 (25.0)	0.573	0.0
DAS28, mean (SD)	2.90 (1.50)	2.86 (1.49)	3.07 (1.54)	0.076	15.8
CVD history, *n* (%)	89 (6.0)	68 (5.5)	21 (8.9)	0.063	0
Hypertension, *n* (%)	546 (37.1)	453 (36.7)	93 (39.4)	0.466	0.0
Dyslipidemia, *n* (%)	251 (17.1)	210 (17.0)	41 (17.4)	0.961	0.0
Diabetes, *n* (%)	136 (9.2)	116 (9.4)	20 (8.5)	0.749	0.0
Treatments					
Previous use of statins; *n* (%)	251 (17.1)	210 (17.0)	41 (17.4)	0.961	0
Corticosteroids; *n* (%)	400 (27.2)	347 (28.1)	53 (22.5)	0.090	0.0
CsDMARD; *n* (%)	1,101 (74.8)	924 (74.8)	177 (75.0)	1.000	
Targeted synthetic or bDMARD treatment				0.114	0.0
None (%)	455 (30.9)	391 (31.6)	64 (27.1)	0.194	
Abatacept (%)	142 (9.6)	122 (9.9)	20 (8.5)	0.586	
IL6 (%)	165 (11.2)	137 (11.1)	28 (11.9)	0.814	
TNFi (%)	390 (26.5)	310 (25.1)	80 (33.9)	0.006	
JAKi (%)	36 (2.4)	31 (2.5)	5 (2.1)	0.901	
Other (%)	284 (19.3)	245 (19.8)	39 (16.5)	0.277	
Previous targeted synthetic bDMARD (%)				0.616	0.0
0	202 (13.7)	171 (13.8)	31 (13.1)		
1	350 (23.8)	287 (23.2)	63 (26.7)		
2	307 (20.9)	256 (20.7)	51 (21.6)		
3+	613 (41.6)	522 (42.2)	91 (38.6)		
Outcomes during follow-up					
Median follow-up duration (IQR)	4.42 (2.09–7.32)	4.51 (2.07–7.39)	3.93 (2.15–6.97)	0.198	
MACE (%)	35 (2.4)	27 (2.2)	8 (3.4)	0.379	
Non-fatal myocardial infarction (%)	11 (0.7)	10 (0.8)	1 (0.4)	0.828	
Non-fatal stroke (%)	18 (1.2)	15 (1.2)	3 (1.3)	1.000	
Cardiovascular death (%)	6 (0.4)	2 (0.2)	4 (1.7)	0.005	
ECR (%)	31 (2.1)	29 (2.3)	2 (0.8)	0.222	
Death by any cause (%)	78 (5.3)	62 (5.0)	16 (6.8)	0.342	0.0
Other causes of death (%)					
Cancer	7 (0.5)	6 (0.5)	1 (0.4)	1.000	
Infection	7 (0.5)	6 (0.5)	1 (0.4)	1.000	
Respiratory disease	10 (0.7)	10 (0.8)	0 (0.0)	0.340	
Other causes	48 (3.3)	38 (3.1)	10 (4.2)	0.470	
Biochemistry					
Total cholesterol (mmol/L), mean (SD)	5.36 (1.17)	5.40 (1.17)	5.13 (1.20)	0.001	0.1
HDL cholesterol (mmol/L)	1.56 (0.48)	1.58 (0.49)	1.48 (0.46)	0.002	0.1
LDL cholesterol (mmol/L)	1.57 (0.87)	1.59 (0.86)	1.49 (0.94)	0.095	0.1
Triglycerides (mmol/L)	3.06 (1.01)	3.07 (1.01)	3.00 (1.00)	0.353	3.5
Non-HDL cholesterol (mmol/L)	3.79 (1.12)	3.82 (1.13)	3.65 (1.09)	0.038	0.1
Total cholesterol to HDL ratio	3.44 (2.79–4.35)	3.45 (2.77–4.34)	3.38 (2.82–4.35)	0.611	0.1
Triglycerides to HDL ratio	1.96 (1.42–2.66)	1.96 (1.41–2.64)	1.91 (1.50–2.71)	0.295	3.5
Median Hs-CRP, mg/L (IQR)	2.08 (0.79–5.41)	2.04 (0.82–5.49)	2.22 (0.69–4.99)	0.648	0.0
Median NT-proBNP (pg/ml) (IQR)	81.8 (45.2–154.0)	81.4 (45.3–150.7)	85.7 (43.1–164.5)	0.507	0.7
Hs-cTnT (ng/L) (IQR)	4.40 (0.00–8.66)	4.40 (0.00–8.60)	4.38 (0.00–9.15)	0.832	0.7
RF + ACPA seropositivity, *n* (%)	1,050 (78.1)	876 (77.9)	174 (79.5)	0.667	8.7

### Associations with study outcomes

During 4.4 years of median follow-up, 2.4% (35/1,472) of patients with RA presented the primary outcome of MACE (6 CV deaths, 11 non-fatal MI, and 18 non-fatal stroke) and 2.1% (31/1,472) of patients presented the secondary outcome consisting of ECR. The crude incidence rate for MACE was 5.2 events per 1,000 patient years (95% CI: 3.6–7.3), 1.6 events per 1,000 patient years for non-fatal MI (95% CI: 0.8–2.9), 2.7 events per 1,000 patients for non-fatal stroke (95% CI: 1.6–4.2), and 0.9 events per 1,000 patients for CV deaths (95% CI: 0.3–1.9). As shown in Table 2, patients with RA with MACE during follow-up were more likely to be men, smokers, had a longer follow-up, and tended to be more likely treated with conventional DMARD at baseline. They also had lower HDL levels, higher LDL levels, higher median hs-cTnT, and higher atherogenic indexes compared to patients without MACE during follow-up. No other difference was noted, especially regarding rheumatoid factor (RF) and anti-citrullinated protein (CCP) seropositivity, which were not associated with MACE (Table 2). These differences were no longer significant in adjusted analyses (see Supplementary Table 1).

**Table 2:** Sociodemographic and biochemical measures stratified according to the occurrence of MACE during the follow-up.

Number of patients	Overall	No MACE	MACE during FU	*p*	Missing data in %
	1,472	1,437	35		
Sociodemographic					
Age, mean (SD)	58.12 (13.25)	58.02 (13.31)	62.35 (9.69)	0.769	0.0
Male sex, *n* (%)	372 (25.3)	350 (24.4)	22 (62.9)	<0.001	0.0
Disease duration, mean (SD)	10.68 (9.97)	10.72 (9.98)	9.21 (9.37)	0.269	0.9
Smoker, *n* (%)	393 (26.7)	375 (26.1)	18 (51.4)	0.001	0.0
DAS28, mean (SD)	2.90 (1.50)	2.90 (1.50)	2.70 (1.37)	0.966	15.8
CVD history, *n* (%)	89 (6.0)	86 (6.0)	3 (8.6)	0.253	0.0
Hypertension, *n* (%)	546 (37.1)	528 (36.7)	18 (51.4)	0.110	0.0
Dyslipidemia, *n* (%)	251 (17.1)	244 (17.0)	7 (20.0)	0.809	0.0
Diabetes, *n* (%)	136 (9.2)	131 (9.1)	5 (14.3)	0.454	0.0
Treatments					
Previous use of statins, *n* (%)	251 (17.1)	244 (17.0)	7 (20.0)	0.809	0
Corticosteroids, *n* (%)	400 (27.2)	387 (26.9)	13 (37.1)	0.250	0.0
CsDMARD, *n* (%)	1,101 (74.8)	1,069 (74.4)	32 (91.4)	0.036	0.0
Targeted synthetic or bDMARD treatment (%)				0.930	0.0
None (%)	455 (30.9)	443 (30.8)	12 (34.3)	0.801	0.0
Abatacept (%)	142 (9.6)	140 (9.7)	2 (5.7)	0.612	0.0
IL6 (%)	165 (11.2)	162 (11.3)	3 (8.6)	0.818	0.0
TNFi (%)	390 (26.5)	379 (26.4)	11 (31.4)	0.634	0.0
JAKi (%)	36 (2.4)	36 (2.5)	0 (0.0)	0.693	
Other (%)	284 (19.3)	277 (19.3)	7 (20.0)	1.000	0.0
Previous targeted synthetic or bDMARD (%)				0.611	0.0
0	202 (13.7)	199 (13.8)	3 (8.6)		
1	350 (23.8)	339 (23.6)	11 (31.4)		
2	307 (20.9)	299 (20.8)	8 (22.9)		
3+	613 (41.6)	600 (41.8)	13 (37.1)		
Median follow-up duration (IQR)	4.42 (2.09–7.32)	4.35 (2.05–7.28)	6.57 (4.41, 8.68)	0.001	0.0
Biochemistry					
Total cholesterol (mmol/L), mean (SD)	5.36 (1.17)	5.36 (1.16)	5.28 (1.53)	0.686	0.1
HDL cholesterol (mmol/L)	1.56 (0.48)	1.57 (0.48)	1.31 (0.50)	0.001	0.1
LDL cholesterol (mmol/L)	1.57 (0.87)	1.56 (0.85)	1.99 (1.46)	0.004	0.1
Triglycerides (mmol/L)	3.06 (1.01)	3.06 (1.00)	3.22 (1.30)	0.347	3.5
Non-HDL cholesterol (mmol/L)	3.79 (1.12)	3.79 (1.12)	3.97 (1.38)	0.340	0.1
Median total cholesterol to HDL ratio	3.44 (2.79–4.35)	3.42 (2.77–4.32)	4.28 (3.37–5.08)	0.001	0.1
Median triglycerides to HDL ratio	1.96 (1.42–2.66)	1.95 (1.42–2.64)	2.52 (1.71–3.43)	0.006	3.5
Median Hs-CRP (mg/L) (IQR)	2.08 (0.79–5.41)	2.06 (0.81–5.39)	2.63 (0.71–8.33)	0.548	0.0
Median NT-proBNP (pg/ml) (IQR)	81.80 (45.20–154.00)	82.00 (45.30–154.75)	73.30 (34.40–145.00)	0.595	0.7
Median Hs-cTnT (ng/L) (IQR)	4.40 (0.00–8.66)	4.32 (0.00–8.59)	5.82 (3.89–10.36)	0.014	0.7
AAA1, OD (IQR)	0.40 (0.28)	0.40 (0.28)	0.48 (0.31)	0.082	0.0
AAA1 seropositivity, *n* (%)	236 (16.0)	228 (15.9)	8 (22.9)	0.379	0.0
RF + ACPA seropositivity, *n* (%)	1,050 (78.1)	1,024 (78.2)	26 (76.5)	0.979	8.7

C-statistics indicated that AAA1 levels were significant predictors of MACE (AUC: 0.60, 95% CI: 0.51–0.68, *p* = 0.03) and of CV deaths (AUC: 0.77, 95% CI: 0.57–0.98, *p* = 0.01). Comparable AUCs were observed for atherogenic indexes (see Supplementary Table 2). MACE occurred in similar proportions between AAA1 seronegative and AAA1 seropositive patients (2.2% vs. 3.4%, *p* = 0.38), and so did non-fatal MI and strokes (0.8% vs. 0.4%, *p* = 0.83, and 1.2% vs. 1.3%, *p* = 1). However, CV deaths were less frequent among AAA1 seronegative patients compared to seropositive patients (0.2% vs. 1.7%; *p* = 0.005).

**Figure 1:**
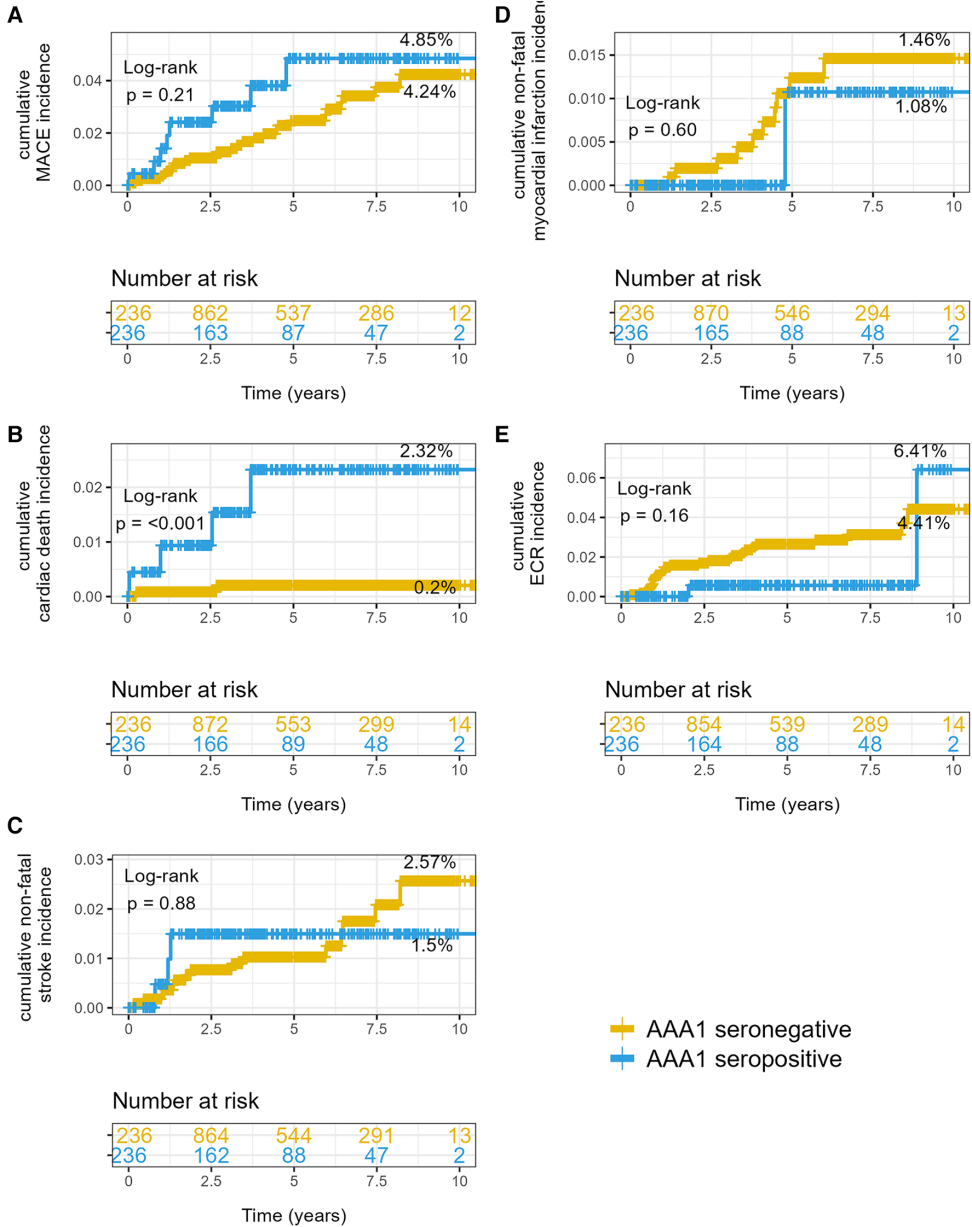
Cumulative incidences of hazards according to AAA1 seropositivity status. (**A**) MACE, (**B**) cardiovascular deaths, (**C**) non-fatal stroke, and (**D**) non-fatal myocardial infarctions. (**E**) Elective coronary revascularization. *p*-values are given by log-rank test. The percentage is the final cumulative incidence rate in each stratum.

Kaplan–Meier analyses illustrated in Figure 1 yielded cumulative incidences for MACE of 4.85% in AAA1 seropositive individuals vs. 4.24% in AAA1 seronegative patients (*p* = 0.21). When we examined the different MACE components, the cumulative incidences in AAA1 seropositive vs. seronegative individuals were 2.32% vs. 0.2% (*p* < 0.001) for CV deaths, 1.49% vs. 2.60% for non-fatal strokes (*p* = 0.89), and 1.09% vs. 1.48% for non-fatal MI (*p* = 0.60), respectively.

The Poisson regression analysis (see Table 3) confirmed these results. The IRR was increased by 1.6 (95% CI: 0.7–3.5, *p* = 0.22) for MACE when comparing AAA1 seropositive and AAA1 seronegative patients. The IRR remained unchanged after adjustment for other potential confounding factors. For CV deaths, the unadjusted IRR was 11.1 (95% CI: 2.1–80.3) when comparing AAA1 seropositive and AAA1 seronegative patients; after adjustment, the IRR was 6.8 (95% CI: 1.3–49.6). AAA1 seropositivity did not predict significant changes in the incidence of MI, stroke, or other CV events, as detailed in Table 3. In addition, we explored the impact of AAA1 levels as a continuous exposure variable, rather than binary seropositivity. In the adjusted model, each AAA1 increment of 1 AU level was associated with a significant 2.5-fold adjusted increase (95% CI: 1.07–5.4, *p* = 0.03) of MACE incidence, and with a significant 8.7-fold adjusted increase (95% CI: 1.3–60.2, *p* = 0.02) in the incidence of CV deaths. A Cox regression analysis provided similar results (see Supplementary Table 3).

**Table 3:** Incidence rate ratio according to AAA1 seropositivity status and AAA1 continuous levels.

Outcome	Exposure	Univariable	Matched analysis
MACE	AAA1 seropositivity	1.64 (0.70–3.46), *p* = 0.22	1.43 (0.61–3.00), *p* = 0.38
CV deaths	AAA1 seropositivity	11.13 (2.17–80.31), *p* = 0.005	6.87 (1.34–49.59), *p* = 0.03
Non-fatal MI	AAA1 seropositivity	0.55 (0.03–2.90), *p* = 0.57	0.59 (0.03–3.09), *p* = 0.62
Non-fatal stroke	AAA1 seropositivity	1.12 (0.26–3.39), *p* = 0.86	1.86 (0.43–5.63), *p* = 0.33
ECR	AAA1 seropositivity	0.38 (0.06–1.26), *p* = 0.18	0.36 (0.06–1.19), *p* = 0.16
MACE	AAA1 (AU)	2.42 (0.88–5.49), *p* = 0.06	2.52 (1.07–5.43), *p* = 0.03
CV deaths	AAA1 (AU)	8.70 (1.65–29.92), *p* = 0.002	8.71 (1.29–60.23), *p* = 0.02
Non-fatal MI	AAA1 (AU)	2.01 (0.25–8.50), *p* = 0.43	1.38 (0.13–8.95), *p* = 0.76
Non-fatal stroke	AAA1 (AU)	1.14 (0.18–4.55), *p* = 0.87	1.37 (0.29–3.97), *p* = 0.63
ECR	AAA1 (AU)	0.40 (0.07–1.66), *p* = 0.26	0.73 (0.15–2.92), *p* = 0.67

Exposure was AAA1 seropositivity and AU increase of AAA1 (AAA1 OD) for all the study endpoints (MACE, CV death, non-fatal MI, non-fatal stroke, and ECR). Adjusted Poisson analyses were performed for each endpoint on a matched dataset based on a propensity score including age, disease duration, gender, smoking, NT-proBNP, hs-cTnT, DAS28, HDL cholesterol, and the atherogenic indexes (total cholesterol to HDL ratio and triglyceride to HDL ratio). Incidence rate ratios are reported with 95% confidence intervals in parenthesis.

Finally, at the predefined and validated AAA1 cut-off value of 0.64 AU, the SE, SP, NPV, and PPV for MACE were 22.8% (95% CI: 11.4–37.1), 84.2% (95% CI: 82.2–86.0), 97.8% (95% CI: 97.5–98.2), and 3.4% (95% CI: 1.5–5.6), respectively. The LR+ was 1.45 (95% CI: 0.64–2.44) and the LR− was 0.92 (95% CI: 0.74–1.07).

For CV deaths, the SE, SP, NPV, and PPV were 66.7% (95% CI: 24.1–94.0), 84.2 (95% CI: 82.0–85.8), 99.8% (95% CI: 99.3–100), and 1.7% (95% CI: 0.5–4.5), respectively. LR+ was 4.23 (95% CI: 2.37–7.54) and the LR− was 0.39 (95% CI: 0.12–1.22).

For non-fatal myocardial infarction, the SE, SP, NPV, and PPV were 9.4% (95% CI: 0.0–27.3), 83.9% (95% CI: 81.8–85.8), 99.2% (95% CI: 99.1–99.4), and 0.4% (95% CI: 0.0–2.7), respectively. The LR+ was 0.59 (95% CI: 0.02–1.79) and the LR− was 1.08 (95% CI: 0.86–1.21).

For non-fatal stroke, the SE, SP, NPV, and PPV were 16.8% (95% CI: 0.0–33.3), 83.9% (95% CI: 82.0–85.9), 98.8% (95% CI: 98.6–99.0), and 1.3% (95% CI: 0.0–2.6), respectively. The LR+ was 1.05 (95% CI: 0.02–2.17) and the LR− was 0.99 (95% CI: 0.76–2.17).

### Associations with features of the lipid paradox in RA

Spearman analyses indicated that both atherogenic indexes correlated strongly together (r = 0.95; *p* < 0.0001) and modestly with C-reactive protein high-sensitivity (Hs-CRP) (r = −0.11; *p* < 0.02 for both). AAA1 were modestly and inversely correlated with total cholesterol (r = −0.17; *p* < 0.0001), HDL cholesterol (r = −0.06; *p* = 0.03), LDL cholesterol (r = −0.11; *p* < 0.0001), non-HDL cholesterol (−0.13; *p* < 0.0001), and triglyceride levels (−0.09; *p* < 0.001), while no correlation was found with total cholesterol to HDL ratio (r = −0.04; *p* = 0.10) or triglycerides to HDL ratio (r = −0.02; *p* = 0.38).

## Discussion

In this large representative multicentric RA cohort, the AAA1 seropositivity rate was 16% and of the same order of magnitude than that retrieved previously in RA (14, 34), but lower compared to the 43% observed in systemic lupus erythematosus (SLE). In this cohort, AAA1 seropositivity was significantly associated with male sex, corroborating what has been described in RA previously (14, 34), and contrasting with the absence of such an association in non-RA settings (18–24), including SLE (38). This suggests the existence of a possible RA-specific AAA1 sex association, warranting further studies. While AAA1 seropositivity is associated with anti-phospholipid antibody seropositivity in SLE (38), AAA1 were not found to be associated with RF and/or anti-CCP antibodies in RA, confirming previous reports (14, 34). Because all these different autoantibodies share common expression of quantitative trait loci in the Fc gamma receptor like 3 gene (21, 39), the absence of associations between RA-specific autoantibodies and AAA1 suggests that other factors than genetic predisposition may drive the AAA1 occurrence in patients with RA.

The key finding of the present study is that a single AAA1 measure independently predicts subsequent risk of incident CV events, which in this study were mostly driven by CV deaths. Corroborating previous reports, including large multicenter prospective studies performed in non-RA settings (14, 18–24, 34), our results suggest that these autoantibodies may potentially better predict fatal CV events than non-fatal CV ones in RA. Further work is required to confirm or refute this assumption. Of note, the MACE incidence of 5.2 events per 1,000 patients retrieved in our study was overall comparable to others (8, 11, 14, 17, 40–42), especially when one considers variations of threefold, depending upon countries and events reporting methodology (11). The relatively high proportion of patients on biologic disease-modifying anti-rheumatic drug (bDMARD) therapy at baseline could have lowered the incidence of MACE when compared to a bDMARD naïve population (43). Even if our study design does not allow us to ascertain causality, the strength of the associations retrieved between AAA1 and CV deaths were at least equivalent or even higher than what has been retrieved for established CV risk factors in RA (41). As the data from *in vitro* or animal models show, AAA1s elicit a pro-inflammatory/pro-atherogenic response through toll-like receptor (TLR) 2/4/CD14 complex signaling (23, 26–30), and impair HDL antioxidant function through paraoxonase-1 inhibition (44), current hypotheses propose that AAA1 increase CV risk by sustaining a low-grade chronic pro-inflammatory milieu (16). In terms of potential clinical applications, the close to optimal NPVs observed for the primary outcome at the redefined AAA1 cut-off (>97%, with lower limits of 95% CI >97% as well) indicates that these autoantibodies could be used as an initial step of a CV screening strategy in RA to rule out individuals at increased risk. On the other hand, assessing those autoantibodies for rule-in purposes is very unlikely. Although our analysis shows that prediction capacity of CV deaths by AAA1 is robust to the adjustment for various known CV risks factors, we could not assess the ability of AAA1 to provide incremental prognostic information over usual CV risk score in RA (see the Limitations discussed below). Therefore, the present results need to be replicated together with other CV risk stratification tools before any clinical recommendations can be made.

The fact that AAA1 seropositivity was associated with lower total and HDL cholesterol levels is in line with previous observations in non-RA settings (19–22). Replicating such a pattern in RA indicates that AAA1 may potentially play a role in the RA lipid paradox, characterized by lower total and LDL cholesterol levels but increased CV risk (45, 46). This hypothesis is substantiated by *in vitro* analyses indicating that AAA1 upregulates LDL-receptor expression and LDL uptake in macrophages, followed by increased esterified intracellular pools of cholesterol through acyl coenzyme A cholesterol acyltransferase activation, in parallel to the increase of cellular cholesterol synthesis through 3-hydroxy-3-methylglutaryl CoA reductase activation in a TLR2/4/CD14-dependent manner (26). The hypothesis that inflammatory stimuli favors shifting of extracellular lipids toward intracellular pools, leading to generate foam cells (26), provides an appealing explanation of the lipid paradox in RA in AAA1 seropositive individuals. Because the lipid paradox plays a key role in the underestimation of cardiovascular disease (CVD) risk in RA by common stratification tools (45), understanding the role of these autoantibodies is important for our understanding of CVD development in immune-mediated diseases.

The present study has some limitations. First, there were a limited number of events during follow-up, which prevented us from performing more extensive adjusted analyses and resulted in large confidence intervals of the estimators. The reason for such a low event number is mainly a limited follow-up. Second, we could not generate previously reported scores (Framingham Risk Score, SCORE2, QRISK) (8, 10–14), mostly due to the fact that arterial blood pressure was not recorded in the SCQM CRF. Knowing whether AAA1 enhances the performance of these previously reported CV risk stratification scores warrants additional studies but has been demonstrated by the same team in another cohort previously (14). A further limitation is that we could not evaluate the link between those antibodies and HDL function in the present study. As HDL function is gaining momentum in RA (46) and because AAA1 may promote the loss of HDL antioxidant properties (44), such field would be worth further exploration. Due to the limited number of samples and the fact that no standardized method exists regarding HDL function evaluation, we could not explore this aspect in top of the association between AAA1 and the lipid paradox in RA. Finally, as a biomarker phase 3 study designed to validate previous findings on the CV predictive ability of AAA1, we did not measure other autoantibodies of potential interest in RA, such as RF, anti-CCP, or anti-HDL antibodies (15, 17, 30, 31, 33). To our knowledge, this is the first large-scale confirmation of the CV prognostic value of AAA1, which are, to date, the most extensively characterized autoantibodies of the anti-HDL family from an analytical, biological, and clinical standpoint (14, 16, 18–30, 32).

In conclusion, this study performed at a large multicentric scale indicates that AAA1 represent independent predictors of CV death in RA and are associated with the lipid paradox susceptible to contribute to the underestimation of CV risk in these patients. However, their potential incremental value over current risk stratification tools remains to be demonstrated before any clinical recommendations can be made.

## Data Availability

Dataset can be shared upon reasonable request, provided that the SCQM registry grants permission for the research project.

## References

[1] Avina-Zubieta JA Thomas J Sadatsafavi M Lehman AJ Lacaille D. Risk of incident cardiovascular events in patients with rheumatoid arthritis: a meta-analysis of observational studies. Ann Rheum Dis. (2012) 71(9):1524–9. 10.1136/annrheumdis-2011-200726 22425941

[2] Conrad N Verbeke G Molenberghs G Goetschalckx L Callender T Cambridge G Autoimmune diseases and cardiovascular risk: a population-based study on 19 autoimmune diseases and 12 cardiovascular diseases in 22 million individuals in the UK. Lancet. (2022) 400(10354):733–43. 10.1016/S0140-6736(22)01349-6 36041475

[3] Skeoch S Bruce IN. Atherosclerosis in rheumatoid arthritis: is it all about inflammation? Nat Rev Rheumatol. (2015) 11(7):390–400. 10.1038/nrrheum.2015.40 25825281

[4] Arts EEA Popa C Den Broeder AA Semb AG Toms T Kitas GD Performance of four current risk algorithms in predicting cardiovascular events in patients with early rheumatoid arthritis. Ann Rheum Dis. (2015) 74(4):668–74. 10.1136/annrheumdis-2013-204024 24389293

[5] Agca R Heslinga SC Rollefstad S Heslinga M McInnes B Peters MJL EULAR recommendations for cardiovascular disease risk management in patients with rheumatoid arthritis and other forms of inflammatory joint disorders: 2015/2016 update. Ann Rheum Dis. (2017) 76(1):17–28. 10.1136/annrheumdis-2016-209775 27697765

[6] Robertson J Peters MJ McInnes IB Sattar N. Changes in lipid levels with inflammation and therapy in RA: a maturing paradigm. Nat Rev Rheumatol. (2013) 9(9):513–23. 10.1038/nrrheum.2013.91 23774906

[7] Ajeganova S Humphreys JH Verheul MK van Steenbergen HW van Nies JAB Hafström I Anticitrullinated protein antibodies and rheumatoid factor are associated with increased mortality but with different causes of death in patients with rheumatoid arthritis: a longitudinal study in three European cohorts. Ann Rheum Dis. (2016) 75(11):1924–32. 10.1136/annrheumdis-2015-208579 26757747

[8] Arts EEA Popa CD Den Broeder AA Donders R Sandoo A Toms T Prediction of cardiovascular risk in rheumatoid arthritis: performance of original and adapted SCORE algorithms. Ann Rheum Dis. (2016) 75(4):674–80. 10.1136/annrheumdis-2014-206879 25691119

[9] Corrales A González-Juanatey C Peiró ME Blanco R Llorca J González-Gay MA. Carotid ultrasound is useful for the cardiovascular risk stratification of patients with rheumatoid arthritis: results of a population-based study. Ann Rheum Dis. (2014) 73(4):722–7. 10.1136/annrheumdis-2012-203101 23505241

[10] Corrales A Vegas-Revenga N Atienza-Mateo B Corrales-Selaya C Prieto-Peña D Rueda-Gotor J Combined use of QRISK3 and SCORE as predictors of carotid plaques in patients with rheumatoid arthritis. Rheumatology. (2021) 60(6):2801–7. 10.1093/rheumatology/keaa718 33249513

[11] Crowson CS Rollefstad S Kitas GD van Riel PLCM Gabriel SE Semb AG Challenges of developing a cardiovascular risk calculator for patients with rheumatoid arthritis. PLoS One. (2017) 12(3):e0174656. 10.1371/journal.pone.0174656 28334012 PMC5363942

[12] Curtis JR Xie FL Crowson CS Sasso EH Hitraya E Chin CL Derivation and internal validation of a multi-biomarker-based cardiovascular disease risk prediction score for rheumatoid arthritis patients. Arthritis Res Ther. (2020) 22(1):282. doi: 10.1186/s13075-020-02355-0 10.1186/s13075-020-02355-0 PMC7718706 33276814

[13] Ferraz-Amaro I Corrales A Atienza-Mateo B Vegas-Revenga N Prieto-Pena D Sanchez-Martin J SCORE2 assessment in the calculation of cardiovascular risk in patients with rheumatoid arthritis. Diagnostics. (2021) 11(12):2363. 10.3390/diagnostics11122363 34943599 PMC8700102

[14] Finckh A Courvoisier DS Pagano S Bas S Chevallier-Ruggeri P Hochstrasser D Evaluation of cardiovascular risk in patients with rheumatoid arthritis: do cardiovascular biomarkers offer added predictive ability over established clinical risk scores? Arthrit Care Res. (2012) 64(6):817–25. 10.1002/acr.21631 22302385

[15] Hejblum BP Cui J Lahey LJ Cagan A Sparks JA Sokolove J Association between anti-citrullinated fibrinogen antibodies and coronary artery disease in rheumatoid arthritis. Arthrit Care Res. (2018) 70(7):1113–7. 10.1002/acr.23444 PMC5891393 28992379

[16] Satta N Frias MA Vuilleumier N Pagano S. Humoral immunity against HDL particle: a new perspective in cardiovascular diseases? Curr Pharm Des. (2019) 25(29):3128–46. 10.2174/1381612825666190830164917 31470782

[17] Wahlin B Innala L Magnusson S Moller B Smedby T Rantapaa-Dahlqvist S Performance of the expanded cardiovascular risk prediction score for rheumatoid arthritis is not superior to the ACC/AHA risk calculator. J Rheumatol. (2019) 46(2):130–7. 10.3899/jrheum.171008 30275258

[18] Antiochos P Marques-Vidal P Virzi J Pagano S Satta N Bastardot F Association between anti-apolipoprotein A-1 antibodies and cardiovascular disease in the general population results from the CoLaus study. Thromb Haemostasis. (2016) 116(4):764–71. doi: 10.1160/Th16-03-0248 27384400 10.1160/TH16-03-0248

[19] Anderson JLC Pagano S Virzi J Dullaart RPE Annema W Kuipers F Autoantibodies to apolipoprotein A-1 as independent predictors of cardiovascular mortality in renal transplant recipients. J Clin Med. (2019) 8(7):948. doi: 10.3390/jcm8070948 10.3390/jcm8070948 PMC6679113 31261925

[20] Antiochos P Marques-Vidal P Virzi J Pagano S Satta N Hartley O Impact of CD14 polymorphisms on anti-apolipoprotein A-1 IgG-related coronary artery disease prediction in the general population. Arterioscl Throm Vas. (2017) 37(12):2342. 10.1161/ATVBAHA.117.309602 29074586

[21] Antiochos P Marques-Vidal P Virzi J Pagano S Satta N Hartley O Anti-apolipoprotein A-1 IgG predict all-cause mortality and are associated with Fc receptor-like 3 polymorphisms. Front Immunol. (2017) 8:437. 10.3389/fimmu.2017.00437 28458671 PMC5394854

[22] Vuilleumier N Pagano S Combescure C Gencer B Virzi J Räber L Non-linear relationship between anti-apolipoprotein A-1 IgGs and cardiovascular outcomes in patients with acute coronary syndromes. J Clin Med. (2019) 8(7):1002. 10.3390/jcm8071002 PMC6679072 31324073

[23] Montecucco F Vuilleumier N Pagano S Lenglet S Bertolotto M Braunersreuther V Anti-apolipoprotein A-1 auto-antibodies are active mediators of atherosclerotic plaque vulnerability. Eur Heart J. (2011) 32(4):412–21. 10.1093/eurheartj/ehq521 21224292

[24] Wick PA Mombelli A Pagano S Moren X Giannopoulou C Mach F Anti-apolipoprotein A-1 autoantibodies as biomarker for atherosclerosis burden in patients with periodontitis. J Periodontal Res. (2013) 48(3):350–6. 10.1111/jre.12014 23050768

[25] Mannic T Satta N Pagano S Python M Virzi J Montecucco F CD14 as a mediator of the mineralocorticoid receptor-dependent anti-apolipoprotein A-1 IgG chronotropic effect on cardiomyocytes. Endocrinology. (2015) 156(12):4707–19. 10.1210/en.2015-1605 26393305

[26] Pagano S Magenta A D’Agostino M Martino F Barillà F Satta N Anti-apo A-1 IgGs in familial hypercholesterolemia display paradoxical associations with lipid profile and promote foam cell formation. J Clin Med. (2019) 8(12):2035. 10.3390/jcm8122035 PMC6947407 31766415

[27] Pagano S Satta N Werling D Offord V de Moerloose P Charbonney E Anti-apolipoprotein A-1 IgG in patients with myocardial infarction promotes inflammation through TLR2/CD14 complex. J Intern Med. (2012) 272(4):344–57. 10.1111/j.1365-2796.2012.02530.x 22329401

[28] Vuilleumier N Rossier MF Pagano S Python M Charbonney E Nkoulou R Anti-apolipoprotein A-1 IgG as an independent cardiovascular prognostic marker affecting basal heart rate in myocardial infarction. Eur Heart J. (2010) 31(7):815–23. 10.1093/eurheartj/ehq055 20176799

[29] Montecucco F Braunersreuther V Burger F Lenglet S Pelli G Carbone F Anti-apo A-1 auto-antibodies increase mouse atherosclerotic plaque vulnerability, myocardial necrosis and mortality triggering TLR2 and TLR4. Thromb Haemostasis. (2015) 114(2):410–22. doi: 10.1160/Th14-12-1039 25879306 10.1160/TH14-12-1039

[30] Pagano S Carbone F Burger F Roth A Bertolotto M Pane B Anti-apolipoprotein A-1 auto-antibodies as active modulators of atherothrombosis. Thromb Haemostasis. (2016) 116(3):554–64. doi: 10.1160/Th16-03-0229 27356567 10.1160/TH16-03-0229

[31] Rodríguez-Carrio J Alperi-López M López P Ballina-García FJ Abal F Suárez A. Antibodies to high-density lipoproteins are associated with inflammation and cardiovascular disease in rheumatoid arthritis patients. Transl Res. (2015) 166(6):529–39. 10.1016/j.trsl.2015.07.004 26279255

[32] Rodriguez-Carrio J Alperi-Lopez M Lopez P Perez-alvarez AI Robinson GA Alonso-Castro S Humoral responses against HDL are linked to lipoprotein traits, atherosclerosis, inflammation and pathogenic pathways during early arthritis stages. Rheumatology. (2023) 62(8):2898–907. 10.1093/rheumatology/kead009 36617161

[33] Rodríguez-Carrio J López-Mejías R Alperi-López M López P Ballina-García FJ González-Gay MA Paraoxonase 1 activity is modulated by the rs662 polymorphism and IgG anti-high-density lipoprotein antibodies in patients with rheumatoid arthritis potential implications for cardiovascular disease. Arthritis Rheumatol. (2016) 68(6):1367–76. 10.1002/art.39609 26815637

[34] Vuilleumier N Bas S Pagano S Montecucco F Guerne PA Finckh A Anti-apolipoprotein A-1 IgG predicts major cardiovascular events in patients with rheumatoid arthritis. Arthritis Rheum-Us. (2010) 62(9):2640–50. 10.1002/art.27546 20506304

[35] Lauper K Courvoisier DS Chevallier P Finckh A Gabay C. Incidence and prevalence of major adverse cardiovascular events in rheumatoid arthritis, psoriatic arthritis, and axial spondyloarthritis. Arthrit Care Res. (2018) 70(12):1756–63. doi: 10.1002/acr.23567 10.1002/acr.23567 29609199

[36] Riek M Scherer A Möller B Ciurea A von Mühlenen I Gabay C Serious infection risk of tofacitinib compared to biologics in patients with rheumatoid arthritis treated in routine clinical care. Sci Rep Uk. (2023) 13(1):17776. 10.1038/s41598-023-44841-w PMC10584888 37853058

[37] Team RC. R: a language and environment for statistical computing. R Found Stat Comput. (2019). https://www.R-project.org

[38] Nigolian H Ribi C Courvoisier DS Pagano S Alvarez M Trendelenburg M Anti-apolipoprotein A-1 autoantibodies correlate with disease activity in systemic lupus erythematosus. Rheumatology. (2020) 59(3):534–44. doi: 10.1093/rheumatology/kez306 31377780 10.1093/rheumatology/kez306

[39] Kochi Y Yamada R Suzuki A Harley JB Shirasawa S Sawada T A functional variant in, encoding Fc receptor-like 3, is associated with rheumatoid arthritis and several autoimmunities. Nat Genet. (2005) 37(5):478–85. 10.1038/ng1540 15838509 PMC1362949

[40] Argnani L Zanetti A Carrara G Silvagni E Guerrini G Zambon A Rheumatoid arthritis and cardiovascular risk: retrospective matched-cohort analysis based on the RECORD study of the Italian society for rheumatology. Front Med Lausanne. (2021) 8:745601. doi: 10.3389/fmed.2021.745601 34676228 10.3389/fmed.2021.745601 PMC8523847

[41] Nikiphorou E de Lusignan S Mallen CD Khavandi K Bedarida G Buckley CD Cardiovascular risk factors and outcomes in early rheumatoid arthritis: a population-based study. Heart. (2020) 106(20):1566. 10.1136/heartjnl-2019-316193 32209618 PMC7525791

[42] Turesson C Jarenros A Jacobsson L. Increased incidence of cardiovascular disease in patients with rheumatoid arthritis: results from a community based study. Ann Rheum Dis. (2004) 63(8):952–5. 10.1136/ard.2003.018101 15051620 PMC1755101

[43] Singh S Fumery M Singh AG Singh N Prokop LJ Dulai PS Comparative risk of cardiovascular events with biologic and synthetic disease-modifying antirheumatic drugs in patients with rheumatoid arthritis: a systematic review and meta-analysis. Arthritis Care Res. (2020) 72(4):561–76. 10.1002/acr.23875 PMC6745288 30875456

[44] Batuca JR Amaral MC Favas C Paula FS Ames PRJ Papoila AL Extended-release niacin increases antiapolipoprotein A-1 antibodies that block the antioxidant effect of high-density lipoprotein-cholesterol: the EXPLORE clinical trial. Brit J Clin Pharmaco. (2017) 83(5):1002–10. 10.1111/bcp.13198 PMC5401980 27891663

[45] Mackey RH Kuller LH Moreland LW. Cardiovascular disease risk in patients with rheumatic diseases. Clin Geriatr Med. (2017) 33(1):105–77. 10.1016/j.cger.2016.08.008 27886692

[46] Weber B He ZL Yang N Playford MP Weisenfeld D Iannaccone C Divergence of cardiovascular biomarkers of lipids and subclinical myocardial injury among rheumatoid arthritis patients with increased inflammation. Arthritis Rheumatol. (2021) 73(6):970–9. 10.1002/art.41613 33615723 PMC9707860

