# Supplementary material for: Anti-apolipoprotein A-1 IgG, incident cardiovascular events, and lipid paradox in rheumatoid arthritis
Source: Front Cardiovasc Med. 2024 May 20;11:1386192. doi: 10.3389/fcvm.2024.1386192 (PMC11144907; doi:10.3389/fcvm.2024.1386192)
Supplement: Supplementary file 1 [file Datasheet1.pdf]

Supplementary table 1 : Socio-demographic and baseline biochemical characteristics according the occurrence of MACE during the follow-up on the matched dataset.

	Overall	No MACE	MACE during FU	p
N	735	700	35	
**Socio-demographic**				
Age (mean (SD))	62.18 (11.77)	62.17 (11.87)	62.35 (9.69)	0.930
Male Gender; n (%)	337 (45.9)	315 (45.0)	22 (62.9)	0.058
Disease duration (mean (SD))	10.00 (9.82)	10.04 (9.84)	9.21 (9.37)	0.623
Smoker; n (%)	296 (40.3)	278 (39.7)	18 (51.4)	0.229
DAS 28 (mean (SD))	2.81 (1.49)	2.81 (1.49)	2.70 (1.37)	0.672
CVD History ; n (%)	64 (8.7)	61 (8.7)	3 (8.6)	1.000
Hypertension; n (%)	344 (46.8)	326 (46.6)	18 (51.4)	0.698
Dyslipidemia; n (%)	156 (21.2)	149 (21.3)	7 (20.0)	1.000
Diabetes; n (%)	87 (11.8)	82 (11.7)	5 (14.3)	0.848
**Treatments**				
Corticosteroids ; n (%)	202 (27.5)	189 (27.0)	13 (37.1)	0.264
treatment (%)				0.873
none (%)	61 (8.3)	59 (8.4)	2 (5.7)	
Abatacept (%)	185 (25.2)	174 (24.9)	11 (31.4)	
il6 (%)	72 (9.8)	69 (9.9)	3 (8.6)	
TNFi (%)	17 (2.3)	17 (2.4)	0 (0.0)	
JAKi (%)	243 (33.1)	231 (33.0)	12 (34.3)	
other (%)	157 (21.4)	150 (21.4)	7 (20.0)	
Previous bDMARD (%)				0.616
0	123 (16.7)	120 (17.1)	3 (8.6)	
1	201 (27.3)	190 (27.1)	11 (31.4)	
2	156 (21.2)	148 (21.1)	8 (22.9)	
3+	255 (34.7)	242 (34.6)	13 (37.1)	
Median Follow-up duration (IQR)	6.05 (3.47-8.07)	6.03 (3.47-8.04)	6.57 (4.41- 8.68)	0.150
**Biochemistry**				
Total chol., mmol/l (mean (SD))	5.38 (1.26)	5.39 (1.25)	5.28 (1.53)	0.617
HDL chol., mmol/l	1.40 (0.41)	1.40 (0.40)	1.31 (0.50)	0.185
LDL chol., mmol/l	1.78 (0.94)	1.76 (0.91)	1.99 (1.46)	0.170
Triglycerides, mmol/l	3.19 (1.08)	3.19 (1.07)	3.22 (1.30)	0.847
Non-HDL chol., mmol/l	3.99 (1.19)	3.99 (1.19)	3.97 (1.38)	0.938
Median Total Chol. to HDL ratio (IQR)	3.94 (3.16-4.76)	3.93 (3.15-4.75)	4.28 (3.37-5.08)	0.188
Median Triglycerides to HDL ratio (IQR)	2.29 (1.70, 3.01)	2.29 (1.69-2.97)	2.52 (1.71- 3.43)	0.274
Median Hs-CRP, mg/l (IQR)	2.47 (1.00-5.81)	2.46 (1.01- 5.77)	2.63 (0.71-8.33)	0.978
Median NT-proBNP, pg/ml (IQR)	88.10 (45.45-188.00)	89.30 (46.00_190.00)	73.30 (34.40- 145.00)	0.315
Median Hs-cTnT, ng/l (IQR)	5.86 (3.42-10.45)	5.86 (3.40-10.40)	5.82 (3.89-10.36)	0.645
AAA1, OD (IQR)	0.32 [0.20, 0.50]	0.32 [0.20, 0.49]	0.37 [0.28, 0.62]	0.026
AAA1 seropositivity ; n (%)	120 (16.3)	112 (16.0)	8 (22.9)	0.403

	Overall	No MACE	MACE during FU	p
RF + ACPA seropositivity ; n (%)	515 (77.6)	489 (77.6)	26 (76.5)	1.000

Table abbreviations:

CVD: cardiovascular disease, ECR: Elective coronary revascularization, HDL: High-density lipoprotein; LDL: Low density lipoprotein; RF: rheumatoid factor, ACPA: Anti–citrullinated protein antibody; NT-proBNP : N-terminal pro-brain natriuretic peptide; Hs-cTnT: high sensitive cardiac troponin T; Hs-CRP: C-Reactive Protein High-Sensitivity; bDMARD: biologic Disease-modifying anti-rheumatic drug

Supplementary table 2: C-statistics of different baseline biomarkers to predict study endpoints

	MACE	CV deaths	MI	Stroke	ECR
Total cholesterol AUC (95%CI)	0.52 (0.41-0.63) p=0.66	0.54 (0.22-0.86) p=0.64	0.52 (0.30-0.74) p=0.59	0.55 (0.35-0.75) p=0.32	0.53 (0.43-0.63) p=0.28
HDL cholesterol AUC (95%CI)	0.66 (0.56-0.76) p=1.00	0.73 (0.50-0.97) p=0.98	0.75 (0.58-0.93) p=1.00	0.67 (0.48-0.85) p=0.049	0.58 (0.49-0.67) p=0.94
LDL cholesterol AUC (95%CI)	0.63 (0.55-0.71) p=0.004	0.62 (0.46-0.78) p=0.15	0.64 (0.46-0.81) p=0.06	0.52 (0.32-0.72) p=0.42	0.57 (0.46-0.68) p=0.09
Triglycerides AUC (95%CI)	0.53 (0.41-0.64) p=0.31	0.53 (0.25-0.80) p=0.41	0.55 (0.32-0.77) p=0.30	0.52 (0.32-0.73) p=0.59	0.53 (0.43-0.64) p=0.27
Non-HDL cholesterol AUC (95%CI)	0.53 (0.42-0.64) p=0.26	0.55 (0.27-0.82) p=0.35	0.56 (0.35-0.76) p=0.25	0.53 (0.33-0.73) p=0.62	0.57 (0.47-0.67) p=0.11
Total Chol./HDL ratio AUC (95%CI)	0.66 (0.57-0.76) p<0.001	0.76 (0.62-0.90) p=0.01	0.72 (0.55-0.89) p=0.005	0.66 (0.47-0.85) p=0.95	0.59 (0.50-0.69) p=0.04
Triglycerides/HDL ratio AUC (95%CI)	0.64 (0.54-0.74) p=0.003	0.74 (0.59-0.89) p=0.02	0.71 (0.52-0.91) p=0.010	0.67 (0.48-0.86) p=0.96	0.57 (0.47-0.67) p=0.10
Hs-CRP AUC (95%CI)	0.53 (0.42-0.64) p=0.27	0.66 (0.41-0.91) p=0.09	0.58 (0.38-0.77) p=0.19	0.62 (0.43-0.82) p=0.89	0.54 (0.43-0.64) p=0.25
NT-proBNP AUC (95%CI)	0.53 (0.42-0.64) p=0.70	0.55 (0.30-0.80) p=0.33	0.70 (0.51-0.88) p=0.99	0.65 (0.46-0.83) p=0.07	0.60 (0.49-0.71) p=0.03
Hs-cTnT AUC (95%CI)	0.62 (0.54-0.70) p=0.007	0.57 (0.45-0.70) p=0.26	0.57 (0.42-0.72) p=0.22	0.63 (0.44-0.82) p=0.10	0.61 (0.50-0.72) p=0.02
AAA1 AUC (95%CI)	0.60 (0.51-0.68) p=0.03	0.77 (0.57-0.98) p=0.01	0.61 (0.48-0.74) p=0.11	0.65 (0.46-0.84) p=0.94	0.46 (0.35-0.56) p=0.78

Supplementary table 3: Hazard ratios (95% confidence interval) according to AAA1 seropositivity and AAA1 continuous levels.

outcome	exposure	univariable	Matched analysis
MACE	AAA1 seropositivity	1.64 (0.75-3.62) p=0.22	1.82 (0.83-4.01) p=0.14
CV deaths	AAA1 seropositivity	10.68 (1.96-58.31) p=0.006	5.17 (0.94-28.37) p=0.06
MI	AAA1 seropositivity	0.58 (0.07-4.55) p=0.61	0.56 (0.07-4.36) p=0.58
stroke	AAA1 seropositivity	1.10 (0.32-3.80) p=0.88	1.20 (0.35-4.14) p=0.78
ECR	AAA1 seropositivity	0.37 (0.09-1.57) p=0.18	0.40 (0.10-1.68) p=0.21
MACE	AAA1, AU	2.39 (0.96-5.91) p=0.06	2.47 (1.06-5.77) p=0.04
CV deaths	AAA1, AU	7.84 (1.99-30.97) p=0.003	4.29 (0.86-21.56) p=0.08
MI	AAA1, AU	2.08 (0.37-11.86) p=0.41	2.05 (0.31-13.65) p=0.46
stroke	AAA1, AU	1.13 (0.23-5.66) p=0.88	1.33 (0.27-6.45) p=0.73
ECR	AAA1, AU	0.41 (0.08-1.97) p=0.26	0.52 (0.11-2.40) p=0.40

Table legend:

Exposure was AAA1 seropositivity and arbitrary unity (AU) increase of AAA1 for all the study endpoints (Major Adverse Cardiovascular Event MACE; Cardiovascular CV death, Non-fatal Myocardial Infarction MI, Non-fatal stroke, and Elective Coronary Revascularization ECR). Adjusted Cox analyses were performed for each endpoint on a matched dataset based on a propensity score including age, disease duration, gender, smoking, NT-proBNP, hs-cTnT, DAS28, HDL cholesterol, and the atherogenic indexes (total cholesterol to HDL ratio and triglyceride to HDL ratio). Hazard ratios are reported with 95% confidence intervals in parenthesis.
